# Young peoples’ interface with providers of contraceptive care: a simulated client study in two Ugandan districts

**DOI:** 10.1186/s40834-016-0027-0

**Published:** 2016-09-07

**Authors:** Gorrette Nalwadda, Florence Mirembe, Josaphat Byamugisha, Nazarius M. Tumwesigye, Elisabeth Faxelid

**Affiliations:** 1 Department of Nursing, College of Health Sciences Makerere University, P.O Box 7072, Kampala, Uganda; 2 Department of Obstetrics and Gynecology, College of Health Sciences Makerere University, P.O Box 7072, Kampala, Uganda; 3 Department of Epidemiology and Biostatistics, School of Public Health, College Health Sciences Makerere University, Kampala, Uganda; 4 Division of Global Health (IHCAR), Department of Public Health Sciences, Karolinska Institutet, Nobels vag 9, 171 76 Stockholm, Sweden

**Keywords:** Contraceptive care, Client-provider interactions, Satisfaction, Young people, Uganda

## Abstract

**Background:**

Young people in Uganda have a large unmet need for modern contraception, and the reasons are unclear. This study describes young peoples’ experiences of contraceptive care, client-provider interactions and its aftermath on choice, access and satisfaction.

**Methods:**

Simulated client method, with 128 encounters with providers in public and private health care facilities was used. Semi-structured narrative debriefing and a structured questionnaire were used to collect data. Content analysis, descriptive and inferential statistics were applied.

**Results:**

Both qualitative and quantitative results highlight favorable reception, provider bias, low client satisfaction and reservations about contraceptive methods. Two thirds of the providers choose a contraceptive method for the client. The clients reported satisfaction with contraceptive services in 29 % of the consultations. Privacy was reported to be observed in 42 % and clients felt respectfully treated in 50 % of the consultations. However, most clients would not recommend the visited facility to others. Client-provider interactions revealed contradictory views on methods to use, whether to first have children, and whether to use contraceptives at all. Younger clients seemed to be treated differently than older clients; contraceptives were provided after a prolonged debate. Inaccurate information about contraceptives was provided and costs were high. Providers conveyed potential adverse effects of contraceptives to young people in a way that indicated providers own fears and doubts.

**Conclusion:**

Young people are not able to exercise their rights to choose, obtain and use contraceptives when needed. Overall satisfaction with the services was rated low and client- provider interactions were often unfavorable.

## Background

Young people enter their reproductive period earlier than preceding generations, and they are inadequately prepared to protect their sexual and reproductive health [1]. Accordingly, the actions young people take to regulate their fertility will determine the size of the future population. The effort to increase access to contraceptives for young people has received global interest. There is an urgent need to address unmet need for contraception and increase access to a full range of voluntary, quality family planning information and services among adolescents and youth [2]. While contraception has been a focus in reproductive health programs in order to prevent unintended pregnancy, HIV, and other STIs for the last five decades, the low use of modern contraceptives is poorly explained.

As the Ugandan population grows, there is parallel growth in contraceptive needs for the increasing group of young people at high risk for unintended pregnancy [3]. The median age of sexual initiation in Uganda is 16 years, and by 20 years of age about 86 % of young persons are sexually active. Young people in Uganda have a large unmet need for modern contraception (34 %) and the reasons are unclear [4].

The pace at which contraceptive use increases has slowed down [2]. In the last 15 years in Uganda, the proportion of married women using oral contraceptive pills has remained low at three percent. Those using condoms have increased from 1 to 2 %, while progestin- only injection users have increased from 3 to 10 percent [5, 6]. The use of implants and Intra Uterine Device (IUD) is only 0.3 % each [6], and has not changed between the two more recent demographic health surveys [7]. Noteworthy, condoms, oral contraceptives, emergency contraceptive pills, progestin- only injections are expected to be provided over-the -counter according to the Uganda national policy [8]. However, young people who do not want to have children still don’t use these methods and continue to have high rates of unintended pregnancy [9]. Research has showed awareness of and positive attitudes to contraception, but the need for contraception is not adequately addressed [7], and little progress has been made to reduce unintended pregnancy particularly among young people [10].

Previous studies have identified distance and cost as constraints to accessing contraceptive services, but clients perception of quality of care might also influence the extent to which these barriers limit access [11, 12]. Research on quality of care has emphasized the importance of client’s perspectives [13, 14]. Studies have shown that even where contraceptive services are available for young people, the service is avoided [15, 16]. The discussion about how the interaction between the clients and the providers influence service use has intensified in recent years [17]. Understanding the client-provider interaction dynamics is important for improving the quality of contraceptive services [18]. This study describes simulated clients experiences of contraceptive care, client-provider interactions and its aftermath on choice, access and satisfaction. It is hoped that the results will provide insights for policy and programs on contraceptive care.

## Methods

The study was conducted in 2010 in two rural districts, Mityana and Mubende, in central Uganda. The two districts were projected to have a population of nearly 1 million people in 2010 [19], a total fertility rate of 8 children per woman [6].

One hundred and thirty health facilities were identified from the Ministry of Health lists of health facilities as has been previously described [20]. Of these, 35 were public, 11 were private not for profit (PNFP), and 84 were private for profit (PFP). Public and PNFP health facilities included two hospitals and 42 health centers while PFP included 20 clinics, four pharmacies, and 60 drug stores. Twenty-one of the facilities were registered as drug stores but operating as clinics. Two facilities did not yield sufficient information (1 public, 1 PNFP) and were not included in the analysis.

### Study design

The simulated client (SC) method was used to study client-provider interactions, provider behavior, and quality of services provided to young people. The method was used to enable collection of first hand data on actual practices of providers from the client’s point of view in a standardized manner [21]. This method limits scrutiny bias by providers, and collects information on observable aspects of client-provider interaction. The SCs, two young men and five young women aged 15–24 were trained by the first author and a sociologist for three days using six case scenarios (Table 1) and role-plays. The SCs were either graduate midwives or had advanced secondary education. The case scenarios were developed by the authors and pilot tested in Kampala district. The scenarios portrayed clients seeking guidance, information and service related to contraception. The female SCs were given one case scenario each while the male SCs were given two to perform throughout the data collection. The SCs visited a particular facility only once, and one client-provider encounter is reported for each of the 128 facilities. Appropriate scenarios for various health facilities and drug stores were used by simulated client during their visits. Drug stores were assesses based on the contraceptive methods they provide such as injections, oral contraceptives, barrier methods and fertility awareness methods as recommended in Uganda guidelines. The SCs were given money to pay for expenses related to the consultations. The first author and field research staff assisted the SC to locate the health facilities.

**Table 1:** Scenerios used by simulated clients

Case 1: Injectable (DMPA)
A married young woman or man aged 20 years with a 2-year-old child. She/his wife has never used contraceptives before but wanted to control fertility because her husband/he has so many other children. She/his wife has never had any pelvic examination before and is on the 3^rd^ day of the menstrual period.
Case 2: Oral contraceptive
A young-looking 16-year-old schoolgirl is having unprotected sex with a steady boyfriend and has no children. She is not sure if she is pregnant or not. She asks the provider for assistance in choosing an oral contraceptive method. She heard from friends that they can prevent pregnancy, nothing more. She has no health conditions (for example, diabetes or high blood pressure).
Case 3*:* Case 3: Implant
A 23-year-old married woman or man, seeking recommendations for a contraceptive method. She/he has two children (aged four and two) and does not wish to have any other child in the next 5 years. She/he knows little about the methods but heard of a method where something can be inserted under the woman’s skin and wants to try it. She/he has not used contraceptives before, and has unprotected sex.
Case 4: Oral contraceptive side effects
A young woman aged 17–19 years is using pills and does not like it any more. She asks the provider whether there is anything she can do about the nausea she has been experiencing since she started taking the pill 2 months ago. She likes the convenience of the pill- it does not interfere with the spontaneity of sex and it is more effective than condoms, which her boyfriend has been using.
Case 5: Condom
A young man is visiting his grandparents and he found a girl he is attracted to in the village, and wants to have sexual intercourse for the first time. He is very scared of making her pregnant and getting HIV. He heard about some contraceptive methods that can be used by a man, and would like to learn about them.
Case 6: Fertility awareness method (FAM)
A married woman or man aged 20 years with two children, one three years and the other 1 year old. She/he has never used contraceptives, doesn’t want to have more children in the near future, but wants to have unprotected sex. The profile requires the client to reject all methods offered except FAM. The client is afraid that the partner may not allow the use of other methods.

### Data collection

The SCs visited contraceptive service health care providers at facilities. The providers were not aware that these clients were involved in research. After each encounter the SC narrated his/her experiences to the first author. These semi-structured narrative debriefing [18], were done as soon as possible after the consultation (mostly within 1 h). The SCs described in as much detail as possible their experiences and feelings about the contraceptive services received and details of their encounter with the provider. These sessions were audio-taped to minimize distortions and notes were also taken. Later, the tape recordings were transcribed. Furthermore, each SC was interviewed after the debrief using a structured questionnaire. The questions related to access to services, waiting time, consultation time, and satisfaction with the services received. The dimensions for measuring client satisfaction were privacy during consultation, respectfulness of health care provider, cleanliness of the facility, and overall satisfaction with the service during the visit. These variables were rated on a scale of 1–5, where 1 was the lowest and 5 was the highest score. Due to lack of variety of the responses, simulated clients’ ratings on dimensions of satisfaction were reduced to a three point format for example “satisfied”, “fairly satisfied”, and “not satisfied” for the analysis. The categories were; 1-2 not satisfied, three fairly satisfied and 4-5 satisfied. Data were also collected on health care provider and facility background characteristics. In addition, data on indicators for assessing quality of care were obtained. These results are reported in another research paper [5].

### Data management and analysis

Quantitative data was entered into a computer using EPIDATA V.3 and was later exported and analyzed using STATA V.11. Frequency distribution of background characteristics of providers and facilities was done. Fisher’s exact chi-square test was carried out to test the significance of the difference in level of client satisfaction by type of facility (public, PNFP, PFP). The test was carried out for each of the four dimension of satisfaction. Similar test was done with waiting and consulting time. Fisher’s exact chi-square test was preferred because of small number of responses in some categories. The level of statistical significance was 0.05.

The qualitative analysis focused on exploring the content of the transcribed narrative accounts. Transcripts from each visit were initially analyzed one by one by the first author. Codes were assigned according to content. These were grouped, and thematic categories constructed according to content for deeper understanding of the SC encounters with the providers [22]. Quotes from SCs were used to enrich the data.

## Results

The background characteristics of facilities and providers are described elsewhere [23]. Visits to 128 facilities are included in the analysis. Two thirds of the providers were nurse/midwives. Other providers were nursing assistant (13 %), medical doctors (10 %), clinical officers (8 %) and others (2 %). The SCs reported that less than a third (26 %) of the health care providers were men. The majority of the facilities provided contraceptive services every day during day time (70 %), while others provided services Monday to Friday (27 %) and some only on specified week days (3 %). The SCs were received well during most of the visits (70 %).

### Choice of contraceptive methods

A large number of the health care providers (71 %) chose or suggested a specific method to the SC. Methods were recommended depending on whether the SC said she/he had children or not. For those with children, the commonly suggested method was progestin-only injection (54 %), followed by pills (17 %), and fertility awareness methods (13 %). Other methods were rarely suggested. The progestin-only injection was also the most suggested method for those with no children (40 %). The condom was mostly suggested for those who did not have children (30 %), (Table 2).

**Table 2:** Contraceptive methods suggested by providers

Contraceptive suggestion	*n *(%)
*Provider suggested contraceptive method N = 125*	
Yes	89 (71.2)
No	36(28.2)
*Suggested methods with/without children N = 89*	
Client with children	52 (58.4)
Client with no children	37 (41.6)
*Specific method suggested to clients with children N = 52*	
Progestin-only injection	28 (53.8)
Oral contraceptive pills	9 (17.3)
Fertility awareness methods (FAM)	7 (13.5)
Condom	1 (2.0)
Intra-uterine device (IUD)	2 (3.8)
Implants	5 (9.6)
*Methods suggested to clients with no children N = 37*	
Progestin-only injection	15 (40.5)
Oral contraceptive pills	4 (10.8)
Fertility awareness methods (FAM)	4 (10.8)
Condom	11 (29.7)
Intra-uterine device (IUD)	1(2.7)
Implants	2 (5.4)

Parity of client is based on case scenario where simulated client were required to say they had children

### Cost of contraceptive services

In more than two thirds (76 %) of the visits the SC paid for the services. The charges ranged from US$ 0.25 (500 UGX) to US$ 10 (20,000 UGX). The payments were for contraceptives (47 %), registration (18 %), consultations (9 %), family planning cards (6 %), blood pressure check (4 %), and medications (16 %) including antibiotics, multivitamin, mebendazole and folic acid. The SC reported paying for services in 7/34 public facilities where services were expected to be free of charge. The cost for long- term contraceptives (IUD and Implants) was said to be US$10 and above, while moon beads, a fertility awareness method, was said to range between $ US 3–5 (6,000–10,000 UGX). Further, the cost of the commonly available methods was on average US$ 0.25 for condom, US$ 0.5 (1,000 UGX) for one cycle of contraceptive pills and US$ 0.75 (1,500 UGX) for three monthly progestin-only injections.

### Waiting and consultation time

The waiting time ranged from zero to 3 h. However, in half of the visits clients waited for less than 5 min (53 %). There was significant difference in waiting time by facility type (*p* < 0.001), clients waited longer during visits in public facilities. The consultations lasted between five and 60 min. In most encounters the consulting time was 10–20 min but there was no significant difference by facility type (Table 3). In 20 % of the visits, the general feeling expressed by the SC was that the consultation was rushed. Long queues on the day of visit were reported in 19 % of the visits and SCs were mixed with either general or antenatal care clients.

**Table 3:** Waiting and consulting time by facility type

Time spent for contraceptive services	Facility type				
	*Total n (%)*	*Public n (%)*	*PNFP n(%)*	*PFP n (%)*	*Exact chi Sq p-value*
*Waiting time in minutes N = 127*					
00	39 (30.7)	7 (20.6)	1 (10.0)	31 (37.3)	χ ^2^ = 30.8
1–4	29 (22.8)	0 (0.0)	4 (40.0)	25 (30.1)	*p* = 0.000
5–50	51 (40.2)	21 (61.8)	4 (40.0)	26 (31.3)	
51–180	8 (6.3)	6 (17.6)	1 (10.0)	1 (1.2)	
*Consulting time in minutes N = 128*					
5–10	42 (32.8)	8 (23.5)	3 (30.0)	31 (36.9)	χ ^2^ = 5.7
11–20	54 (42.2)	15 (44.1)	3 (30.0)	36 (42.8)	*p* = 0.42
21–30	17 (13.3)	5 (14.7)	3 (30.0)	9 (10.7)	
31–60	15 (11.7)	6 (17.6)	1 (10.0)	8 (9.5)	

Categorization of the waiting time and consulting time is informed by the distribution of observations. PNFP-Private not for profit, PFP- private for profit, Note-number of responses are too small in some cells. *P*-value based on exact Fishers test

There was a significant difference in waiting time by facility level across PFP facilities (*p* = 0.001). The waiting time in drugstores was mostly less than 5 min, and longer in clinics (Table 4). There was no significant difference in consulting time across PFP facilities (*p* = 0.32).

**Table 4:** Waiting and consulting time by private for profit facility levels

Time spent for contraceptive services	Facility level				
	*Total n (%)*	*Drugstore n (%)*	*Pharmacy n(%)*	*Clinics n (%)*	*Exact chi Sq p-value*
*Waiting time in minutes N = 83*					
00	31 (37.3)	17 (44.7)	0 (0.0)	14 (34.1)	χ ^2^ = 23.5
1–4	25 (30.1)	12 (31.6)	1 (25.0)	12 (29.3)	*p* = 0.001
5–50	26 (31.3)	9 (23.7)	2 (50.0)	15 (36.6)	
51–180	1 (1.2)	0 (0.0)	1 (25.0)	0 (0.0)	
*Consulting time in minutes N = 83*					
5–10	30 (36.1)	17 (44.7)	1 (25.0)	12 (29.3)	χ ^2^ = 2.27
11–60	53 (63.8)	21 (55.2)	3 (75.0)	29 (70.7)	*p* = 0.32

Categorization of the waiting time and consulting time is informed by the distribution of observations. Note-number of responses are too small in some cells. *P*-value based on exact Fishers test

### Client satisfaction by facility type

Overall, clients reported to be satisfied in 29 % of the consultations. During half of the consultations the SCs said that they were treated with respect. More of the SCs who visited PNFP facilities (8/10) were treated with respect compared to visits in other types of facilities. The SCs said that 16 % of the facilities were dirty at the time of the visit. The SCs considered privacy to be sufficient in 42 % of the consultations. However, privacy during consultations differed by facility type (*p* = 0.04), with privacy mostly observed in PNFP facilities followed by public facilities. The SCs were generally dissatisfied with the services received during 44 % of the visits to public facilities, and during 32 % of the visits to PFP facilities (Table 5). In half of the encounters, SCs stated that based on the services they had received they would not come back to this provider for contraceptive services or recommend the facility to others. Sex of the provider and cost had no effect of overall satisfaction.

**Table 5:** Client satisfaction by facility type

	Health facility type				
Satisfaction	Total *n*(%)	Public *n* (%)	PNFP *n* (%)	PFP *n* (%)	Exact Chi-square, *P*- Value
Cleanness of facility					
Dirty	20 (15.9)	5 (14.7)	0 (0.0)	15 (18.3)	χ ^2^ = 8.4 *p* = 0.08
Fairly clean	42 (33.3)	17 (50.0)	3 (30.0)	22 (26.8)	
Clean	64 (50.8)	12 (35.3)	7 (70.0)	45 (54.9)	
Respectfulness of health staff					
Disrespectful	15 (11.9)	5 (14.7)	0 (0.0)	10 (12.2)	χ ^2^ = 4.6 *p* = 0.41
Fairly respectful	48 (38.1)	12 (35.3)	2 (20.0)	34 (41.4)	
Respectful	63 (50.0)	17 (50.0)	8 (80.0)	38 (46.3)	
Privacy during consultation with provider					
No privacy	38 (30.2)	11 (32.3)	1 (10.0)	26 (31.7)	χ ^2^ = 10.2 *p* = 0.04
Some privacy	35 (27.8)	6 (17.6)	1 (10.0)	28 (34.1)	
Enough privacy	53 (42.0)	17 (50.0)	8 (80.0)	28 (31.1)	
Overall satisfaction with the visit					
Dissatisfied	43 (34.1)	15 (44.1)	2 (20.0)	26 (31.7)	χ ^2^ = 5.2 *p* = 0.30
Fairly satisfied	46 (34.5)	12 (35.3)	6 (60.0)	28 (34.1)	
Satisfied	37 (29.4)	7 (20.6)	2 (20.0)	28 (34.1)	

The score ranges from 1–5; levels of satisfaction were grouped into three to get categories of satisfaction. *P*-values based on Fisher’s exact test Note that the number of responses in some cells is too small. PNFP-private not for profit, and PFP-private for profit

#### Qualitative analysis

The qualitative data from the narrative debriefs provided additional information. Five major themes emerged from the narrative descriptions including; client-provider interactions and perceptions of services received, provider behavior and attitudes, decisions made (methods and restrictions), accuracy of advice and information given, privacy and confidentiality, how providers treated different types of clients. These themes are explored below.

### Client- provider interaction and perceptions of services received

The SCs described hierarchal relationships with the providers. Clients’ narratives indicated that providers frequently used medically inaccurate notions about how conception occurred, and providers also seemed to have fears about the effects of contraception on fertility and menstruation. Discussions between providers and clients focused on negative experiences with contraceptive methods. Providers presented side effects in a manner designed to scare the recipient. Providers also expressed specific fears and doubts about potential or perceived adverse effects of contraceptives on younger people.

*“..Injection causes bleeding, it may be non-stop, others get headaches all the time or on and off menstrual periods, are you ready for that? …. there is tension of taking pills every day and the moment you forget you get pregnant” (SC with a nurse/midwife in public facility).*

There were no standard approaches followed by providers in asking questions and delivering care. Most providers asked about the age of the clients, whether they had children or not, and the number and sex of their children. Some asked about the interest of the partners in having a small family and if co-wives existed. Frequently, the unmarried SCs were asked why they at present didn’t abstain from sex and ask their partners to wait with sex till marriage. Many providers asked questions to rule out an ongoing pregnancy, and to understand what clients knew about contraception.

### Provider behavior and attitudes

Both male and female SCs reported incident of insensitive and disapproving providers. The SCs experienced that most providers would first attend to other clients and some providers even expressed lack of interest in contraceptive care. Some providers were said to be defensive and complaining of shortages of supplies. In terms of communication, unfavorable voice tone and gestures were used by the providers during some consultations. During a few instances (four) visits female SCs experienced sexual harassment by male health care providers.

*“…The provider drew for me a female reproductive organ and explained to me how fertilization takes place. He, however, touched my abdomen without my consent in the way that was inappropriate while he was explaining to me and that made me feel very bad” (SC with Doctor in PNFP facility).*

During some visits, providers gave contraceptive care while doing personal or household chores. While in some of the encounters SCs perceived that providers were too tired and overwhelmed with the large number of clients.

The SCs reported also positive experiences such as providers giving essential information on methods, clearing false beliefs, being friendly, helpful and attempting to address clients’ unique needs. At times providers used unconventional approaches such as giving a written notice for husbands to come and learn about contraceptives.

### Decisions made (methods, restrictions)

The providers often made decision for the clients about methods to use or were of the opinion that the clients should not to use contraception at all. Providers frequently promoted a specific contraceptive method and discouraged other methods. In some cases there were disagreements between the young person and the provider about which method to be chosen. Providers seemed to doubt the clients’ ability to make decisions on contraception. Furthermore, many providers gave the young people contraceptives only after prolonged debate. Requirement for permission from the male partner was a reason for postponing services in some visits. Some providers actively encouraged clients to have children first or have more children before using contraception.

*“…you are lucky if you have a man, first produce more kids before joining such contraceptive methods…oh nurse what can we do?…every contraceptive method is bad- said the lab attendant from the window. The midwife then promoted lactation amenorrhea and withdrawal method. They both laughed at me… the moment I left, they both started talking about me, that they are surprised such a young person wants to use contraceptives… I felt so embarrassed, so small…I left ashamed and disappointed” (SC with a nurse/midwife, in presence of a Lab attendant, PFP facility).*

In some consultations providers expressed beliefs that some methods were not reliable or didn’t work. Commonly providers tried to interest the SCs in methods the provider believed worked or methods that were available at the facility.

*“…Natural family planning methods such as moon beads are good, have no side effects. Since you are a young girl you should use it, but you need to come with your husband. Moon beads to be effective you must be in love to cooperate with partner*” *(SC with nurse/midwife, PFP facility).*

### Accuracy of advice and information given

Mostly providers were said to list the different methods and tell the clients where to find methods not available in the facility. But there was limited or no demonstration of the methods and how each method works. The SCs also reported that limited information was given about follow up and also about STIs prevention. During the discussions the use of IUDs and implants were mostly discouraged for young people. On various occasions’ providers gave inaccurate information, mostly on fertility awareness methods, side effects caused by contraceptives, and how contraceptives work.

*“…About the natural family planning, he told me about the safe days and he said that the 3 days before the periods and the first 4 days after the periods are unsafe while the rest are the safe days. He added that this method is not accurate and he advised me not to use it because two eggs are released one after the other and that if one bursts, the other remains and if one plays sex while in her periods the sperms meet the second egg and one gets pregnant” (SC with a Nurse in PFP Drug shop).*

*“…Pills are good since they prevent pregnancy but since you are a student, and don’t stay with the man, you don’t have to take the pill daily. It is better to take the pill 1 h before having sex” (SC with provider, PFP facility).*

Providers seemed to have limited knowledge on how to manage side effects. Some providers prescribed antibiotics, multivitamin, and other drugs to treat nausea when using contraceptive pills. Furthermore, some providers advised clients to stop the pill altogether if she had nausea. Providers were perceived not to be confident and misconceptions were conveyed as if they were integral part of care in some encounters.

*“…The nurse welcomed me and gave me a seat inside the room. I told her that I have two children and would like to be assisted to avoid pregnancy. She said that family planning methods are not good to use although we have to space children. The nurse said that the injection causes breast and cervical cancer while if the implant gets lost in the arm one can fail to conceive forever, and the pills accumulate and they cause fibroids on the uterus. She, however, admitted that she did not know much about the new methods like the moon bead but recommended that I use safe days and explained the unsafe days as the first seven days after periods while the other days are safe…”(SC with a nurse/midwife in PFP facility).*

### Privacy and confidentiality

Based on SCs narratives, services during some visits were delivered with minimal privacy. Counseling and services were often provided in presence of other clients, in some instances other clients participated in the discussions. According to the SCs, lack of privacy was either non-intentional where facilities were limited by physical space or intentional where space was available but privacy still not provided. Some courteous providers would improvise and move away from other clients to continue the discussion. Even where there were positive experiences with information, privacy and confidentiality were sometimes violated.

*“…I was sitting in a queue with other patients when the nurse came by and asked me what I had come for, I had to tell my story when all other patients were listening that I was going to meet my girl friend for the first time and was scared of pregnancy and getting HIV. She recommended a male condom and taught me briefly how to use it. She also told me to look at the leaflet inside the condom pack to learn more about the instructions. There was no privacy, everyone could hear us. She told me to return in case I encountered problems” (SC with a nurse/midwife PFP facility).*

Interruption from other staff was an additional limitation to privacy and confidentiality. Further, during some of the consultations, there were interruptions by the providers’ family members or friends.

*“…Provider kept moving up and down during consultation, the lab attendant came to the window and stayed all the time, listening and interrupting our discussion or contributing at certain points. I had no time alone with the midwife … I was embarrassed to mention my problems with another person listening and disrupting” (SC visit to a nurse/midwife, in PFP clinic).*

### How providers treated different types of clients

Providers seemed to treat clients differently according to stated age, marital status and sex. Repeatedly, providers discouraged both sexual activity and use of contraceptives for those who identified themselves as unmarried. In a few cases, SCs who stated teen age were reproached or treated harshly by the providers and these clients said they received “parent like” treatment by providers. Comments were often made on clients’ age and appearance. Some SCs reported experiences of manipulation and soliciting for money by the providers.

*“…why do you use family planning, a young girl, see the pill has made you sick, you are going to die....Then he pulled a file and reviewed literature on family planning methods, after reading for about 15 min he said do you have money? Are you willing to pay me 50,000/= for the treatment of the nausea?…You see everything is here in the book I know everything so bring the money, if you don’t have the money there is the exit…” (SC with Clinical officer, PFP clinic).*

Male SCs reported that some providers were excited that men were interested in contraceptive services and male SCs experienced being given special treatment. On the other hand, some providers were pro-women and encouraged use of contraception both for clients with and clients’ with-out children. In a number of cases, those clients who reported to be studying were given contraceptives since they were sexually active.

*“…Provider explained and showed me methods on the chart. She asked me if I had ever done HIV testing. She said that pills are not good for young people like you still studying; they don’t protect against STIs, and also have side effects. She recommended condoms for dual protection” (SC with nurse/midwife, public facility)*

## Discussion

The simulated client method provided important information on the complexities of consultations, provider behavior, and practices when young people request for contraceptive services. The contraceptive consultation is a two-way encounter between a provider and a client. This study highlighted the dynamics involved in contraceptive services provision to young people. Further, the study identified some challenges young people face in accessing contraceptives including cost and unfavorable client-provider relationships. The results also contain some positive practices experienced by both male and female young clients such as good reception and reasonable waiting time.

Results from this study indicate that to a great extent young people experience difficulties to exercise their rights to choose and obtain contraceptives when needed. The results of this study illustrate strong consciously or unconsciously provider bias and control, by way of choosing contraceptive methods for young people. Further, most providers either restricted or refused to offer some contraceptive methods despite the fact that the national reproductive health policy has no such limitations [8]. There is a mismatch between national policy guidelines and actual care provided to young people. This infringes on the rights of young people to choose their preferred method [24], consistent with long standing informed consent governing contraceptive services. Given the biased and improper provider behavior, continued contraceptive use and clients’ possibility to cope with side effects might be difficult, as shown also in other settings [25]. Although the unavailability of a range of contraceptive methods, limits freedom of choice, declining to give a method according to clients’ preference undermines a young persons’ strategy to use contraceptives. Ensuring patient rights has the potential to improve public confidence in healthcare providers’ provision of services.

Our findings illuminated contradictions that arise when young peoples’ contraceptive needs differ from providers’ views about contraceptives. Providers also seemed to treat younger, older, married, and unmarried clients differently. Decision making on behalf of younger persons in contraceptive matters revealed in this study, is ethically and professionally complex since it may contribute to inequalities in health care [17], although providers might in reality want to help the clients with what is available. However, the providers’ assumption that young people are not completely autonomous to make decisions in their best interest is merely speculative. Age alone does not constitute a medical reason for denial of available contraceptive methods to young people [1]. Supporting the providers to realize the intertwined relationship between providers and young clients seeking contraceptive service might help reduce the inevitable errors, injustices and missed opportunities for contraceptive provision to young people.

Based on the simulated clients’ experiences, the services offered were often unsatisfactory. Previous studies have noted that acceptance and use of contraceptives is linked to client satisfaction and quality of care [26]. In the current study, some providers were not respectful and helpful when approached by young people, which was also evident in a previous study where health care providers were interviewed [20]. Good quality of care enhances clients’ satisfaction and their use of services and improves demand [12]. Contraceptive clients are more likely to be satisfied when they are welcomed, treated with respect, and encouraged to ask questions and participate in their health care [27]. Research has also linked client satisfaction to reduced discontinuation rates [28]. In the current study, half of the clients would not recommend the services to prospective clients, which is an indication of clients’ dissatisfaction with services received [29]. Health care provider behavior, respect, and politeness are a powerful predictor of client satisfaction for clients than technical competence of the provider [30]. The same author reported that reduction in waiting time was more important than prolonging consulting time in relation to client satisfaction. In-depth research on contextual determinants of client satisfaction is important.

Contrast to our simulated clients study, exit interviews studies to measure level of client satisfaction revealed that some aspects of quality such as waiting time, price of services, easy to reach the clinic, client satisfaction receive negative response from clients [31]. Exit interviews reveal high levels of satisfaction for example a Iranian study based on exit interviews showed that 49 % of the clients were satisfied with contraceptive service [26]. Whereas, client satisfaction exit interviews are useful to measure aspects of client satisfaction, they are not sufficient to evaluate quality, but could be used alongside other quality evaluation approaches such as simulated clients, direct observation and provider surveys [31].

Clients perceived that rush during service provision and gaps in interaction made facilities and services to be unattractive. Providers of contraceptives cite time constraints as a barrier to offer comprehensive counseling and family planning services to their clients. A previous study using simulated clients showed that relevant information to the client’s choice is optimal when session length is about 14 min [32]. The same author noted that offering a wide range of contraceptive options, discussion of side effects and screening for contraindications is highly correlated with session length. Contraceptive providers should use the available time more resourcefully to assess clients’ needs and better address client method of choice.

Young peoples’ narratives illustrate that providers lack correct and detailed information about contraceptives and reproductive physiology. Information giving behavior seems to be inadequate to facilitate the process of utilization. Correct information on effectiveness, side effects, accurate use, benefits and problems with methods, and follow up is essential to facilitate decision making. Furthermore, people in need of contraception often have question about other issues related to sexuality such as STIs [33]. Such issues were hardly discussed during the consultations. In this study it was evident that providers had limited knowledge about contraceptives but also lacked capacity or interest to review other pertinent issues. They even showed clear misconceptions in some cases, which has also been noted elsewhere [11].

Although most providers had some knowledge of side effects of the methods, the emphasis on side effects of contraceptives was unprecedented. Providers conveyed potential adverse effects of contraceptives to young people in a way that indicated providers own fears, worries and doubts. Such a negative approach has enormous bearing on the initial uptake and continuation of contraceptive use [33].

Contraceptives were mostly offered to those who identified themselves as married. Providers showed negative attitudes to contraceptive use by those not married and those who did not have children. Providers were often reluctant to offer these clients contraceptives. This is probably linked to the strong Ugandan social norm that discourage pre-marital sexual relations and the strong believe in the importance of child bearing [20, 34]. Some providers also discouraged use of IUD and implants by young people. Research indicates that long acting methods can be used by young people and promoting their use among young people has the potential to reduce unintended pregnancy [10, 33]. Private for profit facilities including drug stores provide noticeable contraceptive service to young people, but need appropriate tools to improve their practices. The scope and consequence of this on national policy need to be deliberated.

Cost emerged as an important factor in clients’ access to services. Costs related to consultation, commodities and other services were noted to be high, which might perhaps prevent young adults from obtaining services from private providers [2]. It is important to note that also some public facilities were charging for services, although services are supposed to be free of charge in public facilities. Studies on cost sharing in Uganda have shown that after elimination of user fees at public health facilities in 2001 more people utilized these facilities [29]. Our findings related to cost reflect the risk for inequalities in access to contraceptive care for young persons. Wealth-related inequalities regarding met needs for contraception have also been reported to be on increase in Uganda and Kenya [7].

Our results have ethical, legal (such as restrictions, solicitation) and social implications for healthcare provision. Our study adds evidence that providers practice restrictive non-evidence based practices, which might deter utilization of contraceptives services by young people. To address client-provider disparities, there is need for clear policy direction on contraceptive services in order to improve services quality particularly to young persons in facilities at all levels. It is important to enforce standards of practice to foster behavioral and attitude change in order to build trust and re-vitalize the image of contraceptive services. Structural, behavior, and professional strategies are needed and a focus should be put on midwives a core group in contraceptive care. In addition, to main stream services, targeted services for young people could be a window of opportunity to meet the unique needs of young people such as non-judgmental attitude.

This is a study used young trained SCs. We acknowledge that while these provide good descriptions of the health care provider’s behaviors during the encounters, issues like satisfaction with the services may not be accurately representative of young people.

## Conclusion

During the encounters, providers made contraceptive choices for the clients. The progestin-only injection was the most suggested method. Client-provider interface were dominated by contradictory views on methods to use, whether to first have children, and whether to use contraceptives at all. Clients perceived that provider attitudes and behaviors were negative, younger clients were treated differently than older clients, inaccurate information about contraceptives was provided, and the costs were high and varied. Most clients would not recommend the visited facility to others. Providers conveyed potential adverse effects of contraceptives to young people in a way that indicated providers own fears, worries and doubts. Overall satisfaction with the services was rated low and client- provider interface were often unfavorable. However, mostly clients felt well received and waiting time was favorable. There was positive reception of male clients by most providers. Some providers were pro-women and encouraged use of contraception for all sexually active clients with or with-out children. Many providers asked questions to rule out an ongoing pregnancy. Private for profit facilities including drug stores provide contraceptive service to young people. Intricate balance between improving the quality and volume of services is crucial.

## Abbreviations

HSD, Health Sub District; IUD, intra-uterine device; PFP, private for profit; PNFP, private not for profit; SC, simulated clients; YP, young people
